# Drugs for cardiovascular disease in India: perspectives of pharmaceutical executives and government officials on access and development-a qualitative analysis

**DOI:** 10.1186/s40545-016-0065-7

**Published:** 2016-05-02

**Authors:** Charles Newman, Vamadevan S. Ajay, Ravi Srinivas, Sandeep Bhalla, Dorairaj Prabhakaran, Amitava Banerjee

**Affiliations:** University of Birmingham, Medical School, Birmingham, UK; Centre for Chronic Disease Control, New Delhi, India; Research and Information Systems for Developing Countries (RIS), New Delhi, India; Public Health Foundation of India, New Delhi, India; University of Birmingham Centre for Cardiovascular Sciences, Birmingham, UK; Present address: Farr Institute of Health Informatics Research, University College London, 222 Euston Road, London, NW1 2DA UK; School of Health, University of Central Lancashire, Preston, UK

**Keywords:** Cardiovascular, Stakeholder, Pharmaceutical

## Abstract

**Background:**

India shoulders the greatest global burden of cardiovascular diseases (CVDs), which are the leading cause of mortality worldwide. Drugs are the bedrock of treatment and prevention of CVD. India’s pharmaceutical industry is the third largest, by volume, globally, but access to CVD drugs in India is poor. There is a lack of qualitative data from government and pharmaceutical sectors regarding CVD drug development and access in India.

**Methods:**

By purposive sampling, we recruited either Indian government officials, or pharmaceutical company executives. We conducted a stakeholder analysis via semi-structured, face-to-face interviews in India. Topic guides allow for the exploration of key issues across multiple interviews, along with affording the interviewer the flexibility to examine matters arising from the discussions themselves. After transcription, interviews underwent inductive thematic analysis.

**Results:**

Ten participants were interviewed (Government Officials: *n* = 5, and Pharmaceutical Executives: *n* = 5). Two themes emerged: i) ‘Policy-derived Factors’; ii) ‘Patient- derived Factors’ with three findings. First, both government and pharmaceutical participants felt that the focus of Indian pharma is shifting to more complex, high-quality generics and to new drug development, but production of generic drugs rather than new molecular entities will remain a major activity. Second, current trial regulations in India may restrict India’s potential role in the future development of CVD drugs. Third, it is likely that the Indian government will tighten its intellectual property regime in future, with potentially far-reaching implications on CVD drug development and access.

**Conclusions:**

Our stakeholder analysis provides some support for present patent regulations, whilst suggesting areas for further research in order to inform future policy decisions regarding CVD drug development and availability. Whilst interviewees suggested government policy plays an important role in shaping the industry, a significant force for change was ascribed to patient-derived factors. This suggests a potential role for Indian initiatives that market the unique advantages of its patient population for drug research in influencing national and multinational pharmaceutical companies to undertake CVD drug development in India, rather than simply IP policy-directed factors.

**Electronic supplementary material:**

The online version of this article (doi:10.1186/s40545-016-0065-7) contains supplementary material, which is available to authorized users.

## Background

Cardiovascular Diseases (CVDs) are the principal cause of deaths globally [40, 46, 47, 52] and global spending on CVDs surpasses any other disease [3]. Low- and middle-income countries (LMICs) now shoulder the majority of the global CVD burden [40]; no country more so than India, where ischaemic heart disease (IHD) and stroke were the first and eighth largest causes of years of life lost to death and disability [14, 40]. The economic cost of CVD in India is estimated at $30 billion per annum [32] with future increases forecast [11, 21].

Drugs play an essential role in the prevention and treatment of CVD and its risk factors [3]. Access to drugs plays a significant part in reducing health inequality [12, 50, 51] and is influenced by both affordability and availability [37]. The Indian pharmaceutical industry is now the 3rd largest, by volume, in the world [31]. Furthermore, regardless of recent regulatory changes due to concerns largely about ethics, accountability and compensation [7], India remains one of the world’s most attractive destinations for clinical trials [6]. However, marked inequalities in CVD drug access persist in India [43, 54] and improved access represents a key policy strategy.

Although prevalence studies from India suggested that CVD was more common in urban than rural regions [24], in more developed states of India such as Kerala, the rural–urban differences in cardiometabolic risk factors have largely disappeared and the risk factors are equal or slightly greater in rural subjects [38]. More than 70 % of India’s population lives in rural areas and nearly 40 % of the population is below the poverty line [38], and CVD drugs are less likely to be taken in rural than urban settings [30]. Barriers to CVD drug availability in India include low utilization rates of evidence-based therapies [34], high out-of-pocket expenditure, long duration of therapy and high drug costs relative to income [37, 42]. The poorest are least likely to be able to afford cardiovascular (or any) medications and out of pocket expenditure on healthcare represents the highest proportion of household spending in this group [16, 25]. Over 80 % of CVD patients receive none of the recommended effective drug treatments [25] and low household wealth is the most important determinant. A major portion of overall out of pocket health spending (in excess of 45 %) is for medicines for chronic diseases and this proportion was as high as 64 and 58 % for cases of hypertension and diabetes, respectively [8, 19].

The Indian government spends just 1.2 % of the GDP on the health sector, which is among the lowest in the world [45]. Nearly 846 billion Indian rupees (INR) were spent out of pocket on health care expenses in 2004, amounting to 3.3 % of that year’s gross domestic product (GDP). A major portion of overall out of pocket health spending (in excess of 45 %) was for medicines for NCDs and this proportion was as high as 64 and 58 % for cases of hypertension and diabetes, respectively [19]. As a result of increasing realization of health inequalities in terms of access to healthcare; out-of-pocket expenditure and poverty caused by healthcare expenditure; and an unsustainable national pharmaceutical policy, the Indian government sanctioned a $5.4-billion plan allowing government sector doctors to prescribe generic drugs to patients free of cost [20]. Generic medicines are typically 20 to 90 % cheaper than originator equivalents [17, 49].

Given the importance of CVD, drugs for its treatment and the scale of the Indian pharmaceutical sector, an evidence base is crucial to inform policymakers in India. Qualitative analyses of access to CVD drugs are very limited in low- and low-middle income countries, including India [29]. Government and pharmaceutical companies have been previously identified as the most powerful stakeholders in access to medicines [2]. Therefore we conducted a stakeholder analysis of government officials and pharmaceutical company executives regarding development of and access to CVD drugs in India.

## Aims

Among government officials and pharmaceutical company executives, the aim was conduct a qualitative study to understand the perceptions of the Indian government and pharmaceutical industry about factors affecting the development of new CVD drugs and access to CVD medicines.

## Methods

### Inclusion criteria

Interviewees were eligible for inclusion if they worked at a policy-making level, either in a generation or advisory capacity, in either:

i) The Indian government with reference to CVD pharmaceuticals and/or healthcare (the Government Official sub-group).
ii) A national or multi-national pharmaceutical company, with a base in India, involved in the development of CVD medications (the Pharmaceutical Executive sub-group).

In addition, participants had to be able to communicate fluently in written and spoken English, and to provide informed, written consent.

### Recruitment

Potential participants were contacted via e-mail, using existing contacts. Convenience sampling therefore formed a component of recruitment. However, strict adherence to the inclusion criteria ensured a purposive sampling method, thereby mitigating against the reported inadequacies of a solely convenience sampling approach [36, 41]. Once initial contact was made, informed consent was obtained using documents structured in line with WHO templates [53]. A conservative sample size of 12 participants was initially set [23].

### Interviews

Semi-structured, face-to-face interviews were selected as the most appropriate method of data collection for three reasons. First, this technique allows interviewers to adapt their communication in response to a participant’s behavioural or verbal cue, enabling taxing subjects to be addressed. Second, this method requires a smaller sample size than other qualitative methods [10], which was important given the expected difficulty in recruiting participants from government and pharmaceutical sectors. Third, group-orientated qualitative research methods were felt inappropriate for this study due to the potential for business-sensitive information to arise in the discussions.

Topic guides allow for the exploration of key issues across multiple interviews, along with affording the interviewer the flexibility to examine matters arising from the discussions themselves [5]. Moreover, semi-structuring limits ‘dross rate’, defined by Holloway and Wheeler [26] as information not relevant to the study. Therefore, a topic guide asking open-ended questions regarding three broad issues was constructed as a core component of the semi-structuring of interviews for this study (see Additional file 1). The three broad areas were: (i) role of India in the development of CVD medications both within India and throughout the world; (ii) influence of India in the availability and development of CVD medications in the world pharmaceutical market; and (iii) thoughts/beliefs on the pharmaceutical industry. The wide nature of the points covered in this guide allowed the interviewer the freedom to expand upon emerging themes as they arose during the interviews. Issues were approached from various time perspectives (past, present and future), as an intentional attempt to draw upon the full length of an interviewee’s experience in their field. All interviews were undertaken in February and March 2015. Interviews were undertaken at a location of the interviewee’s choice, in English. Each interview took place in an office environment in New Delhi, Bangalore or Mumbai. Only the interviewer and the participant were in each interview.

**Fig. 1:**
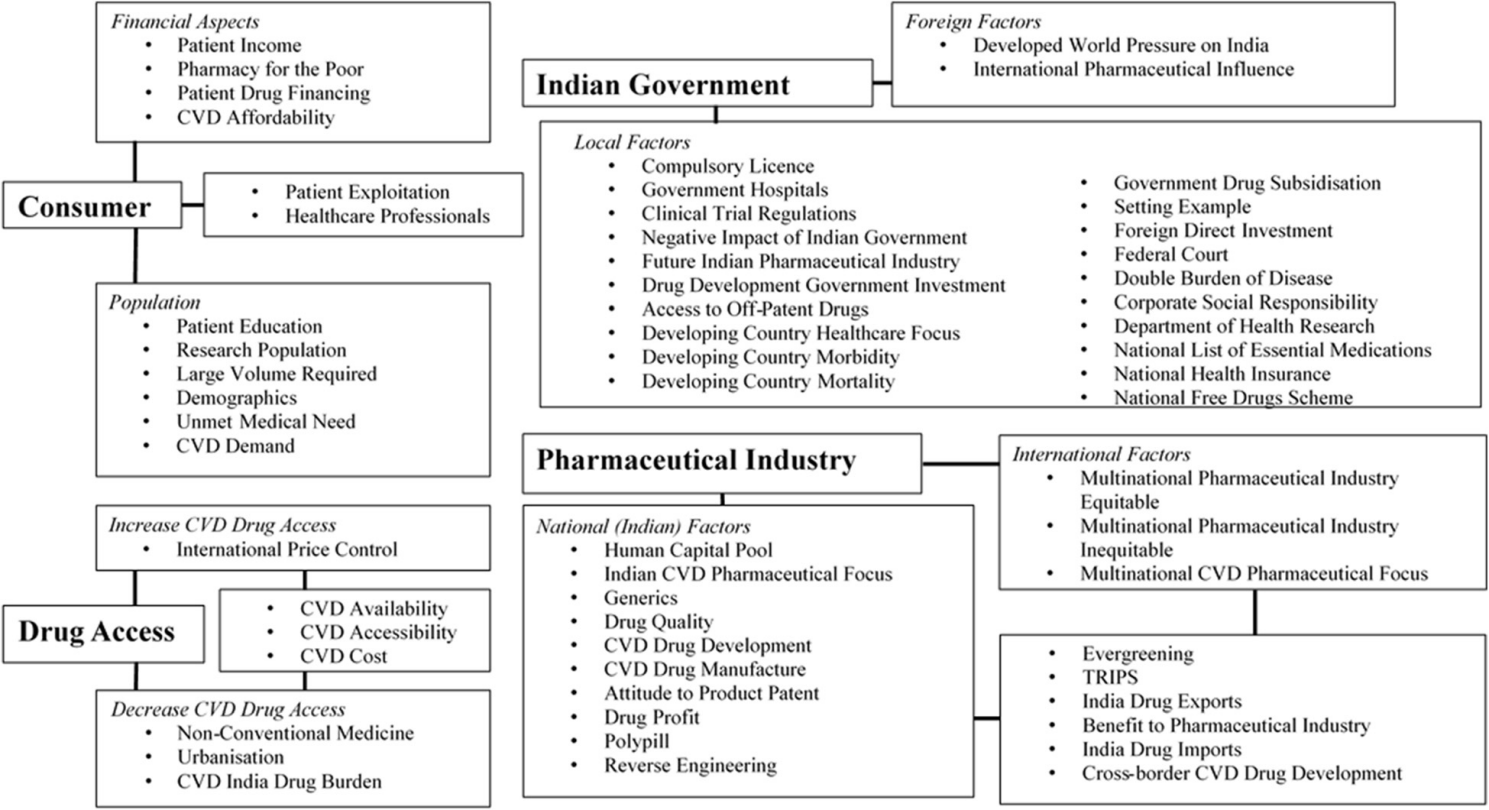
The initial candidate thematic map. Numerous connections were present between the ‘Drug access’ and ‘Consumer’ themes, as well as the ‘Pharmaceutical Industry’ and ‘Indian Government’ themes

**Fig. 2:**
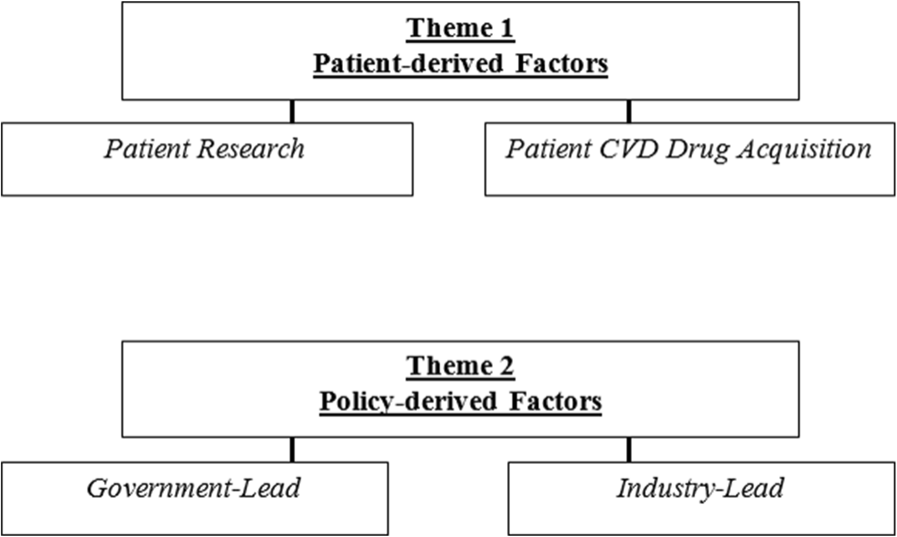
Final thematic map showing the two externally heterogeneous themes discussed in the ‘Results’ section

An audio recording was taken of all interviews. All interviews aimed to last no longer than 30 min. The interviewer would undertake all data analysis and was therefore tasked with transcribing each recording verbatim. In doing this, they became habituated to the data early on, a critical aspect of thematic analysis [9]. Audio software was used to slow the recording during transcription, in an attempt to reduce transcription error rate. The interviewer then completed inductive thematic analysis, as per the distinct 6-stage guidance outlined by Braun and Clarke [9]. Following transcription, the interviewer read through the entire dataset twice to ensure a broad comprehension of the interviews was obtained. All semantic and relevant latent themes were then coded through line-by-line reading of the text, using *NVivo 10* qualitative data analysis software. Semantic grouping of the codes into candidate themes was then undertaken, forming an initial thematic map (see Fig. 1). These themes were then scrutinized for their legitimacy, as outlined by Braun and Clarke [9], and to ensure their internal homogeneity and external heterogeneity was present, as stipulated by Patton [44]. Subsequent adaptation of the first thematic map was required following this review process, resulting in the production of a final thematic map (Fig. 2).

Member checking aims to ensure results presented in qualitative analysis are both credible and reliable, avoiding data misrepresentation [10]. It was felt appropriate to complete this process, due to the potential for complex topics to have arisen during the interviews in this research. Therefore, after completing data analysis, the interviewer e-mailed participants a summary of the initial findings of their interview. Interviewees were asked to check this summary, ensuring that their anonymity had been preserved and nothing had been misinterpreted. No issues of data misrepresentation arose from this process.

Inclusion of a detailed summation of the full analytical process satisfies the assertion that “qualitative research is reliable if one can follow the ‘decision trail’ of the investigative process” [39]. The transparency resulting from the explicit account of the analytical process of this study should, therefore, increase the reliability of these findings, adding rigor to this research.

## Results

**Fig. 3:**
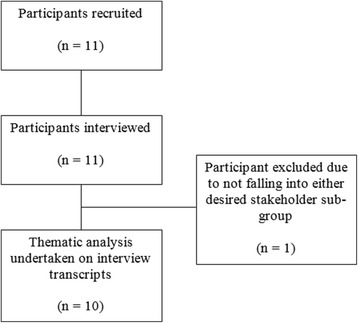
Full participant recruitment flowchart

Eleven interviewees were successfully recruited. Of those recruited, one participant was excluded as it became clear during the interview that they fell into neither one of the stakeholder sub-groups, and were in fact from an entirely clinical background. A final sample of 10 participants was therefore analysed, consisting of a Government Official sub-group (*n* = 5) and Pharmaceutical Executive sub-group (*n* = 5). The full participant recruitment pathway can be seen in Fig. 3. Participant demographic data are shown in Table 1. Actual interview times ranged from 28 to 46 min.

**Table 1:** Participant Demographic Table

Participant ID	Gender	Age (years)	Stakeholder sub-group	Time Working in Respective sub-group (years)
P1	Male	34	Government Official	6
P2	Male	50	Government Official	2.5
P3	Male	74	Government Official	49
P4	Male	53	Government Official	10
P5	Male	52	Government Official	16.5
P6	Female	53	Pharmaceutical Executive	20
P7	Male	46	Pharmaceutical Executive	19
P8	Male	40	Pharmaceutical Executive	13
P9	Male	50	Pharmaceutical Executive	20
P10	Male	52	Pharmaceutical Executive	15

Participant demographics and ID numbers

Responses to the issues outlined in the topic guide (Additional file 1) were broad. However, there were relatively few new viewpoints uncovered in the last interview in each participant sub-group. Two distinct themes emerged: i) Patient-derived Factors; ii) Policy-derived Factors. Both emergent themes and their key topics are presented. Key issues are denoted as sub-headings under their respective theme.

### Theme 1: patient-derived factors

The first distinct theme that emerged from the data contained factors that were either implicitly, or explicitly, linked to the Indian patient population.

#### 1 (a) clinical trial regulations

One participant suggested that the recent tightening of clinical trial regulations in India was a positive factor affecting the role India plays in the development of CVD medicines:

*P1 (Government Official sub-group): “The second thing that has happened which is quite good is tightening of [clinical trial] regulations… the patient population do not get exploited with loose clinical trial regulations.”*

The suggestion made by Participant 1 was not unanimously supported. Other participants, from both stakeholder sub-groups, suggested that present regulations applied too much pressure upon pharmaceutical companies, and were likely to deter drug research and development (R&D) in India:

*P3 (Government Official sub-group): “It’s all negative [referring to the role the Indian government plays in relation to India’s pharmaceutical industry]… there are three areas where we have problems with government… one is the clinical trial regulatory regime…”*

*P6 (Pharmaceutical Executive sub-group): “The regulatory body, that sits in the government… in the last couple of years have come down with regulations related to pharmaceutical industry, which were very detrimental to the progress… on the clinical trial front… it’s not possible to do studies with those regulations in mind…”*

The differences in opinions highlight the complexity of the effects clinical trial regulations have had in India. Tight clinical trial regulations to promote ethical research may be motivated by concern for Indian patients, however, these rules may have a detrimental effect on the Indian pharmaceutical industry, according to participants in both stakeholder sub-groups.

#### 1(b) benefits of undertaking drug development in India

Notwithstanding the conflicting opinion regarding drug regulations in India, participants highlighted numerous benefits to undertaking research in this nation. Firstly, the lack of exposure to previous pharmacological interventions mitigates against confounders present in other research settings, according to Participant 7:

*P7 (Pharmaceutical Executive sub-group): “I think what’s good about India is that there… are a lot of drug naïve patients available… patients who have never been treated with any drugs in the past… so… historically speaking they have a clean slate… and therefore… when you are looking for an effect of a particular drug it’s much simpler because there are no… confounders…”*

This positive factor was not mentioned at all by the Government Official sub-group, and introduced the idea that the Indian patient population may convey a clinical advantage over other nations, with reference to CVD drug development. Participant 7 reported that a further attraction for undertaking CVD medicine development in India stemmed from the large Indian population. They suggested the speed of research could be increased due to ease of patient recruitment:

*P7 (Pharmaceutical Executive sub-group): “Just by sheer population… it’s possible to recruit patients fast… drug development and discovery… can be fast-forwarded… because of… this kind of [large] number of patients that we have.”*

This further example illustrates the importance patient-derived factors have in influencing pharmaceutical executive opinions regarding the best future focus for their business.

#### 1(c) role of the media

Patient-derived factors are not all positive with reference to India’s development of CVD medicines. One participant highlights how unethical research may have exploited patients in the past:

*P10 (Pharmaceutical Executive sub-group): “…and pharmaceutical industry… there are some bad players, and there have been some practices within the industry which were not the most ethical [towards patients] I would say,”*

This interviewee went onto highlight the implicit role the media plays, through incomplete reporting, in tarring the wider reputation of the Indian pharmaceutical industry:

*P10 (Pharmaceutical Executive sub-group): “Having said that [referring to their previous comment regarding unethical research] I think… they [the pharmaceutical industry] want to do the right thing. Unfortunately, I think it is not publicised [to the patients] enough and… it becomes more of… a whipping law… in terms of any of the criticisms that come out…”*

Therefore, whilst patient exploitation is not directly patient-derived, the subsequent detrimental influence of the media on patients is; through the resulting negative opinions of the drug industry. Media was not mentioned by the Government Official sub-group.

#### 1(d) Patient Purchasing Power

Factors grounded in the patient population were commonly mentioned to affect availability of CVD medicines. Firstly, the ability of patients to purchase CVD medicines was highlighted as a significant barrier to CVD drug affordability, by both stakeholder sub-groups:

*P2 (Government Official sub-group): “Access is a function of economic strength of the people… if they… lack purchasing power, even if medicines are available in the villages… they won’t be able to buy that [CVD medicine].”*

*P7 (Pharmaceutical Executive sub-group): “I mean large population of India still lives in its villages… the poverty is just unbelievable… they don’t have money to eat food, let alone have [CVD] medications.”*

The contextualisation of poverty in terms of geographic location by Participant 7 echoes, to some extent, the influence urbanisation has on access to CVD medicines according to another interviewee. Here, difficulties patients encounter in reaching government hospitals, and therefore accessing the CVD medicines and treatments available at these locations, is discussed:

*P6 (Pharmaceutical Executive sub-group): “I mean government hospitals are freely accessible… but even multi-speciality government hospital is very far from many of these rural areas, so many people will reach there only when they are in very bad shape…”*

Patient purchasing power and finances are therefore somewhat linked to a person’s physical location, in relation to the dispenser-point of the CVD drugs.

#### 1(e) patient education

Another frequently mentioned factor which influences CVD drug access is educational level:

*P8 (Pharmaceutical Executive sub-group): “From the patient’s perspective, cardiovascular disease is something which is a… chronic disease and it doesn’t… kill people in a very short time. So I think that itself probably also means that patients are not paying too much of attention to the disease per se,”*

*P5 (Government Official sub-group): “There is a question of [patient] education… better education… better lifestyle, those things ensure… you understand how you need to go for purchasing medicines.”*

Participants from both sub-groups hence spoke of the wider impact patient education has on their awareness to acquire CVD drugs.

#### 1(f) Non-conventional medicine use

A participant in the Government Official sub-group also discussed the use of non-conventional medicine by patients, as a barrier to CVD drug access:

*P5 (Government Official sub-group): “Also it’s [CVD drug access] a question of belief… some of the people… have the traditional way of thinking, so they do not go by the modern [CVD] medicines…”*

Therefore, participants felt that patient education influences CVD drug access, not only through patient motivation to obtain medicine, but also by influencing the type of drugs being used. Patients may be harmed if alternative therapies being used are less clinically effective than conventional CVD drugs.

### Theme 2: policy-derived factors

The second distinct theme that arose from this research regarded issues that were grounded in Indian drug policy.

#### 2(a) Indian pharmaceutical industry focus and its effects

The predominant influence the Indian government has had in the past, according to both stakeholder sub-groups, is in directing the focus of the national pharmaceutical industry. Every participant stated the past, and to a certain extent present, role of the Indian pharmaceutical industry is in the production of off-patent, generic medicines:

*P2 (Government Official sub-group): “We [India] are the generics industry. We are not so much engaged in the new development of… [CVD] drugs.”*

*P6 (Pharmaceutical Executive sub-group): “So I think if you look at Indian pharmaceutical industry, largely it’s generics based.”*

Exploration of India’s current pharmaceutical focus highlighted its role in the provision of low-cost, high quality medicines for the developing world and the West:

*P3 (Government Official sub-group): “India could continue to be a major supplier of quality medicine to the third world countries, and also… in the West…”*

*P8 (Pharmaceutical Executive sub-group): “India has impacted the global pharmaceutical industry a lot by its generic drugs… provided to the global market, whether it is US, Europe, or it is Sub-Saharan countries….”*

Furthermore, participants from both sub-groups touched upon the prospective focus of the Indian CVD pharmaceutical industry. Stakeholders stated the likely future for India will be to produce more complex, high-quality generics and to nurture stronger drug development:

*P3 (Government Official sub-group): “…and within generic, Indian industry’s also moving up the value chain. So, instead of plain generics, they’re moving into a complex, or different chain of generics…”*

*B4 (Government Official sub-group): “The Indian pharmaceutical industry has largely been involved in manufacturing generics… but they do have some new fixed dose combinations and polypills which they developed recently,”*

*P2 (Government Official sub-group): “We [India] are the generics industry. We are not engaged in the new development of… [CVD] drugs, but our… aim is that [to become involved in the development of new CVD drugs].”*

Despite the predicted transition of focus in the Indian industry, participants felt CVD generic medicines will remain a significant player in this market for the foreseeable future:

*P10 (Pharmaceutical Executive sub-group): “…generics will still continue to be the primary driver of the [Indian pharmaceutical] market …”*

One participant suggested the Indian generic industry has cultivated an excessive amount of market competition, to the detriment of drug quality:

*P10 (Pharmaceutical Executive sub-group): “The barriers to entry are so low to make new… generic medicine in India, you have got… 100 brands of atorvastatin now… do you think all 100 brands are going to behave the same way? Probably not… the quality control systems that we invest in, in this facility [referring to their own pharmaceutical company], are much more robust than… some mum and pap making tablets… in a garage…”*

Participants also highlighted how generic CVD medicines increase access, as they are an economically viable option from a prescriber’s perspective:

*P9 (Pharmaceutical Executive sub-group): “Generics lower the cost of treatment, and hence give doctors the choice to prescribe a generic drug, when otherwise they may not have prescribed the innovator drug simply because of cost…”*

In summary, according to participants, the Indian CVD pharmaceutical industry is currently centred on large-scale generic production. This role is likely to change in future to an increased focus on the production of high-quality generics and undertaking more drug R&D.

#### 2(b) Indian human capital

Another significant impact government policy has had on the Indian CVD drug industry, according to this study’s participants, has been the increased availability of scientific human capital:

*P5 (Government Official sub-group): “Our Universities are also good in terms of producing quality professionals, and during the past 10 years a number of pharmacy colleges have been set up… we have good quality of professionals in the field of clinical trial and pharmaceutical technology and pharmaceutical R&D,”*

*P6 (Pharmaceutical Executive sub-group): “One thing which the [Indian] pharmaceutical industry has is lots of very highly skilled manpower…”*

The pool of skilled academics available to undertake advanced drug research is therefore recognised by both stakeholder sub-groups. Furthermore, one participant implied this expert population is likely to grow, rather than decrease, over coming years due to return of trained Indian professionals to the country:

*P8 (Pharmaceutical Executive sub-group): “You can find that a lot of them [Indian men and women] have gone abroad and specialized… in various [pharmaceutical] fields, even got work experience and are willing to come back [to India]…”*

#### 2(c) attitude to product patent

Indian drug policy is also linked to access to CVD medicines according this study’s participants. The international pharmaceutical community would apparently prefer the Indian drug industry to have a more stringent product patent regime. However, a major barrier to this is product patent ‘Evergreening’, defined as a pharmaceutical company’s extension of their monopoly on a drug beyond the usual term permitted by law [18]. Stakeholders form both sub-groups referred to this controversial attitude to patent law:

*P4 (Government Official sub-group): “The multinational companies want a stronger patent regime in India… but the Indian companies don’t want the multinational companies to Evergreen…”*

*P7 (Pharmaceutical Executive sub-group): “You see companies getting greedy… they have some small incremental innovation, and they want now again 20 more years after… the main… patent has… expired… If that can be prevented, then I think the basic intellectual property should belong to those who invest money in it…”*

Despite international pressure for India to tighten its drug patent regime, one participant stated that India’s present attitude to drug patents it not over-lenient, as it conforms to the Trade-Related Aspects of Intellectual Property Rights (TRIPS) agreement:

*P3 (Government Official sub-group): “It’s [India’s product patent regime] not lax, we are conforming to the TRIPS agreement, whatever the standard TRIPS provided, we conform to that…”*

Instead of being down to laxities in India’s approach to intellectual property (IP), this participant suggested the international community were against India’s present IP regime due to their success in producing high-quality generic medicines for the developed world:

*P3 (Government Official sub-group): “Now 80 % of prescriptions in the US are of Indian generics, so the US generic companies are hurt… when you are successful… everyone is going to throw stone at you…”*

Despite this defence of India’s existing attitude to IP, it is likely that the Indian government will tighten regulations in the near future, according to Participant 4. This is due to the influence of other geo-political factors, like the US’s provision of nuclear energy to India:

*P4 (Government Official sub-group): “It’s… rumoured that they [the Indian government] might have an agreement with the US in terms of… tightening the [drug] patent laws… to get other additional benefits… nuclear energy treaty was held hostage to many other things, so they [the Indian government] wanted to get that off the ground,”*

India’s compulsory licensing policy has allowed for past overruling of product patents in order to increase access to specific medicines. This initiative allows a government to develop patented drugs, without the patent-holder’s permission [13]. However, one participant suggested this has detrimentally affected long-term access of novel CVD medicines in India, through mistrust between multinational companies and the Indian government:

*P6 (Pharmaceutical Executive sub-group): “They [the Indian government] have asked the innovator… to give up their patent… you are making it [the CVD drug] available, but then… invalidating their [the innovator company’s] patent in India… that will become a deterrent for new CVD drugs to come to India.”*

Therefore, there are perceived to be numerous external pressures on India attempting to direct the government’s future attitude regarding IP. Altering attitude to IP may, through re-building trust with some multinationals, increase the amount of new CVD products being introduced into the Indian market, thereby potentially improving CVD drug access.

## Discussion

Our study highlights three findings. First, both government and pharmaceutical participants felt that the focus of Indian pharma is shifting to more complex, high-quality generics and to new drug development, but production of generic drugs rather than new molecular entities will remain a major activity. Second, current trial regulations in India may restrict India’s potential role in the future development of CVD drugs. Third, it is likely that the Indian government will tighten its intellectual property regime in future, with potentially far-reaching implications on CVD drug development and access.

The expiry of patents on $60 billion worth of drugs in 2010 suggests that India will remain a significant producer of generics [22]. The Indian pharmaceutical industry’s increasing acquisition of resources to undertake independent R&D make it likely that drug development will gain in importance [27]. Whilst interviewees suggested government policy plays an important role in shaping the industry, a significant force for change was ascribed to patient-derived factors, contrary to current literature [27]. Therefore, there may be a potential role for Indian initiatives that market the unique advantages of its patient population for drug research in influencing national and multinational pharmaceutical companies to undertake CVD drug development in India, rather than simply IP policy-directed factors. It was suggested by one interviewee that rapid patient recruitment represented a major advantage of undertaking research in India. However, 2008 Federal Drug Administration (FDA) data suggest that at inspected clinical trial sites, China may be ahead of India in this regard, with India and China recruiting 8 and 13 participants per recruitment site respectively [33]. There is clearly a need for research into the factors which make India attractive to host CVD drug R&D before policy recommendations can be made, and comparative research with China will be beneficial to inform Indian policy [35].

Although current clinical trial regulations were implicated in restricting Indian CVD drug development, there are two arguments for their existence. First, deviations from ethical research practice have been documented in India previously [48] and therefore, more stringent regulations protect Indian patients from recurrence of such exploitation [35], reiterated by one participant in this study. Second, questions must be raised over the morality of undertaking research with a population that ultimately may not have access to the final product; such as in India where CVD drug availability is an issue. A fine line therefore exists, regarding clinical trial guidelines, between market facilitation and patient protection. However, it is clear that participants felt that the present balance is weighed against the CVD drug development industry. As with other heath policy domains, there is a role for an independent evidence-based appraisal of present clinical trial regulations in India [4] support the use of formal analytical institutes for the of health policy in LMICs, to ensure adequate patient protection, while allowing Indian pharmaceutical development.

Whilst one participant defended India’s current stance, asserting that the government rigorously conform to TRIPS guidelines, other interviewees outlined the external pressure being placed on India to observe to tighter patent regulations. The European Union’s Free Trade Agreement states Europe’s intentions to seek regulations that go beyond the TRIPS agreement in developing countries [15]. Succumbing to such demands may afford India benefits to other sectors, such as energy, according to one stakeholder. Although IP regulations are praised in India for driving the development of a stronger R&D sector within the Indian pharmaceutical industry [27], there has been a lack of technology transfer since India’s signing of the TRIPS agreement in 1992, along with a diminished focus on drug production as per the needs of the national population [1]. However, continued adherence to existing IP regulations may help reinforce trust between the Indian government and multinational drug industry, which is suspicious due to India’s past compulsory licensing [28]. An improved relationship could persuade pharmaceutical companies to make novel CVD medicines readily available on the Indian market, something that they are presently reluctant to do according to this study. Further policy research should investigate the effects of tighter IP control and tighter clinical trial regulations on the scale of drug development in India.

### Limitations

The small sample size of this study is a limitation. However, few novel topics emerged during the final interview of each stakeholder sub-group, suggesting theoretical saturation was being approached. Triangulation, the use of multiple researchers for data analysis [10], was not possible, and would have increased the validity of our findings. Our analysis considered two specific stakeholder subgroups in the Indian context for CVD drugs, and therefore, our findings cannot be generalized to other subgroups, countries or drug areas.

## Conclusion

Among government and pharmaceutical stakeholders, our analysis suggested consensus around three barriers to new CVD drug development and access in India: (i) the prevailing culture, expertise and infrastructure of the drug industry favouring generic production; (ii) strict clinical trial regulations; and (iii) the current IP regime. The role of these different factors on CVD drug development and access in India should be the subject of further research.

### Key messages

1. Both government and pharmaceutical participants felt that the focus of Indian pharma is shifting to more complex, high-quality generics and to new drug development, but production of generic drugs rather than new molecular entities will remain a major activity.
2. Current trial regulations in India may restrict India’s potential role in the future development of CVD drugs.
3. It is likely that the Indian government will tighten its intellectual property regime in future, with potentially far-reaching implications on CVD drug development and access.

### Approval

Ethical approval was awarded from the University of Birmingham BioMedical Science (BSc) Internal Ethics Review Committee (UK), and the Independent Ethics Committee of the Centre for Chronic Disease Control (India).

## Additional file

Additional file 1: Interview Topic Guide. (DOCX 14 kb)
