# Supplementary material for: Drugs for cardiovascular disease in India: perspectives of pharmaceutical executives and government officials on access and development-a qualitative analysis
Source: J Pharm Policy Pract. 2016 May 2;9:16. doi: 10.1186/s40545-016-0065-7 (PMC4852445; doi:10.1186/s40545-016-0065-7)
Supplement: Additional file 1: — Interview Topic Guide. (DOCX 14 kb) [file 40545_2016_65_MOESM1_ESM.docx]

**Additional file 1: Interview Topic Guide**

**The following topic guide was used for interviewing the Government Official stakeholder sub-group:**

1. **Ice-breaker:**
   1. What is your professional background and experience with reference to the pharmaceutical industry?
2. **The role of India in the development of CVD medications both within India and throughout the world:**
   1. General topic questions for overall stakeholder group:
      1. What role does India have in the development of CVD medications?
      2. Has India’s role in the development of CVD medications changed in the recent past?
      3. How would you say India’s role in the development of CVD medications may change in the future?
   2. Topic questions specific for the Government Official sub-group:
      1. What role has the Indian government had in the past regarding drug development?
      2. Has the role of the Indian government changed recently regarding drug development?
      3. What role will the Indian government have in the future regarding drug development?
3. **The influence of India in the availability and development of CVD medications in the world pharmaceutical market:**
   1. General topic questions for overall stakeholder group:
      1. Does India have an influence in the pharmaceutical market?
      2. What was India’s past influence in the pharmaceutical market?
      3. Will India’s influence on the world pharmaceutical market change in the near future?
   2. Topic questions specific for the Government Official sub-group:
      1. Can you think of an example when the Indian government has used its influence in the CVD pharmaceutical market in the past?
      2. Do you think the Indian government will use its influence in the CVD pharmaceutical market in future?
4. **The thoughts/ beliefs on the pharmaceutical industry:**
   1. General topic questions for overall stakeholder group:
      1. What are your opinions on the current pharmaceutical industry?
   2. Topic questions specific for the Government Official sub-group:
      1. Do you think any changes should be made to Indian governmental policy in order to improve the Indian/ world pharmaceutical industry?
      2. What do you think the pharmaceutical industry itself could do to improve availability of medicines?

**The following topic guide was used for interviewing the Pharmaceutical Executive stakeholder sub-group:**

1. **Ice-breaker:**
   1. What is your professional background and experience with reference to the pharmaceutical industry?
2. **The role of India in the development of CVD medications both within India and throughout the world:**
   1. General topic questions for overall stakeholder group:
      1. What role does India have in the development of CVD medications?
      2. Has India’s role in the development of CVD medications changed in the recent past?
      3. How would you say India’s role in the development of CVD medications may change in the future?
   2. Topic questions specific for the Pharmaceutical Executive sub-group:
      1. What role has the pharmaceutical industry had in the past regarding drug development?
      2. Has the role of the pharmaceutical industry changed recently regarding drug development?
      3. What role will the pharmaceutical industry have in the future regarding drug development?
3. **The influence of India in the availability and development of CVD medications in the world pharmaceutical market:**
   1. General topic questions for overall stakeholder group:
4. Does India have an influence in the pharmaceutical market?
5. What was India’s past influence in the pharmaceutical market?
6. Will India’s influence on the world pharmaceutical market change in the near future?
   1. Topic questions specific for the Pharmaceutical Executive sub-group:
      1. Can you think of an example when the pharmaceutical industry has used its influence in the CVD pharmaceutical market in the past?
      2. Will the pharmaceutical industry use its influence in the CVD pharmaceutical market in future?
7. **The thoughts/ beliefs on the pharmaceutical industry:**
   1. General topic questions for overall stakeholder group:
      1. What are your opinions on the current pharmaceutical industry?
   2. Topic questions specific for the Pharmaceutical Executive sub-group:
      1. Do you think any changes should be made to the pharmaceutical industry in order to improve CVD drug availability?
      2. What do you think the Indian government could do to improve access to medicines?
